# The Effect of Pharyngeal Surgery on Positive Airway Pressure Therapy in Obstructive Sleep Apnea: A Meta-Analysis

**DOI:** 10.3390/jcm11216443

**Published:** 2022-10-30

**Authors:** Ki Hwan Kwak, Young Jeong Lee, Jae Yong Lee, Jae Hoon Cho, Ji Ho Choi

**Affiliations:** 1 Department of Otorhinolaryngology-Head and Neck Surgery, Gumi Hospital, Soonchunhyang University College of Medicine, 179, 1gongdan-ro, Gumi 39371, Korea; 2 Department of Otorhinolaryngology-Head and Neck Surgery, Bucheon Hospital, Soonchunhyang University College of Medicine, 170, Jomaru-ro, Bucheon 14584, Korea; 3 Department of Otorhinolaryngology-Head and Neck Surgery, Konkuk University School of Medicine, 120-1, Neungdong-ro, Gwangjin-gu, Seoul 05030, Korea

**Keywords:** continuous positive airway pressure, pharynx, sleep apnea, obstructive

## Abstract

There is controversy about the effect of pharyngeal surgery for obstructive sleep apnea (OSA) on positive airway pressure (PAP) adherence, and the related results of meta-analysis have not yet been available. Therefore, the purpose of this meta-analysis was to assess the effect of pharyngeal OSA surgery on PAP therapy parameters such as optimal pressure levels and usage time. We selected studies investigating optimal PAP levels or usage time before and after pharyngeal OSA surgery, regardless of the study design. Pharyngeal OSA surgery included uvulopalatopharyngoplasty and its variants, tonsillectomy, Pillar implants, radiofrequency ablation, tongue base surgery and its variants, and genioglossus advancement. Studies in which isolated nasal surgery was performed were excluded. The random-effects model was used due to significant heterogeneity among the studies. Nine studies were included in the meta-analysis of optimal PAP levels, and five studies in the meta-analysis of PAP usage time. After pharyngeal OSA surgery, the summed optimal PAP level was significantly decreased (standardized mean difference (SMD), −1.113; 95% confidence interval (CI), −1.667 to −0.559)), and the summed usage time of PAP was significantly increased (SMD, 0.794; 95% CI, 0.259 to 1.329). This study illustrated that pharyngeal OSA surgery lowered optimal PAP levels and enhanced PAP usage time. The results of the meta-analysis contribute to our understanding of the role of pharyngeal OSA surgery in patients with PAP intolerance.

## 1. Introduction

Obstructive sleep apnea (OSA) is a widely prevalent disease characterized by repetitive obstruction of the upper airway, particularly the pharynx, during sleep [1]. Repeated upper airway collapse induces various pathophysiologic conditions, including a hyperactive sympathetic nervous system, intrathoracic pressure swings, sleep fragmentation, intermittent hypoxia, and hypercapnia [2]. These detrimental phenomena may lead to diverse symptoms and critical complications such as excessive daytime sleepiness, impaired concentration, memory loss, impotence, systemic hypertension, diabetes, stroke, decreased quality of life, and an elevated risk of traffic accidents [3]. Therefore, when OSA is suspected, prompt diagnosis and proper therapy are necessary to prevent or manage OSA-related consequences. The treatment options for OSA consist of several methods, including positive airway pressure (PAP), surgical modifications of the upper airway, oral appliances, weight control, and positional therapy [4]. Ultimately, among these therapeutic options, the most appropriate treatment is carried out by considering the patient’s information, such as anatomical structures, polysomnographic results, and treatment preferences [5].

PAP therapy to prevent upper airway obstruction during sleep by providing a pneumatic splint in patients with OSA was first reported by Sullivan et al. [6] in 1981. According to the American Academy of Sleep Medicine (AASM) guidelines, PAP is commonly recommended for the management of patients with OSA, especially moderate-to-severe types and mild-type with comorbidities or significant symptoms [2,7]. The effectiveness of PAP for OSA treatment has been proven through many clinical studies. Compared to no treatment, PAP showed substantial favorable effects in varied aspects, such as excessive daytime sleepiness, diminished sleep-related quality of life, and comorbid systemic hypertension [8,9,10]. To fully achieve the effect of PAP therapy in patients with OSA, an optimal pressure level suitable for the patient’s condition and sufficient usage time play important roles [11].

Surgical modification or reconstruction of the upper airway is usually performed to improve OSA by increasing muscle tension and/or widening the airway space [12]. According to the upper airway anatomy, various surgical techniques for OSA can be classified into nasal surgery (e.g., turbinate surgery, septoplasty, and endoscopic sinus surgery), nasopharyngeal surgery (e.g., nasopharyngeal mass removal and adenoidectomy), oropharyngeal surgery (e.g., uvulopalatopharyngoplasty [UPPP] and its variants, Pillar implants, and tonsillectomy), and hypopharyngeal surgery (e.g., genioglossus advancement and tongue base reduction) [13]. Of these, one or more appropriate surgical techniques are selected and implemented based on the surgical indication and the patient’s anatomical structure.

Some clinical studies have reported that surgical management influenced PAP therapy such as optimal levels and the duration of use [14,15]. Two recent meta-analyses demonstrated that surgical modifications of the upper airway were associated with decreases in optimal PAP levels and improvement in PAP adherence [16,17]. However, one study evaluated the effect of nasal surgery alone on PAP treatment, and the other study investigated the effect of upper airway surgery, including isolated nasal surgery and pharyngeal surgery, on PAP management [16,17]. Furthermore, there is controversy about the effect of pharyngeal OSA surgery on PAP adherence [18]. Therefore, the goal of this study was to ascertain the effect of pharyngeal OSA surgery on PAP therapy, such as optimal pressure levels and the duration of use.

## 2. Materials and Methods

### 2.1. Search Strategy

We performed a comprehensive literature search on the effect of pharyngeal surgery on PAP therapy, including optimal pressure levels and usage time in OSA, using PubMed, SCOPUS, EMBASE, and the Cochrane Library. The keywords included “obstructive sleep apnea,” “sleep-disordered breathing,” “surgery (surgical treatment),” and “continuous positive airway pressure.” The search was conducted on 19 June 2021.

### 2.2. Eligibility Criteria and Study Selection

The studies selected in this review were original articles investigating PAP therapy (optimal pressure levels and/or compliance) before and after pharyngeal OSA surgery, regardless of the study design, which included randomized controlled trials, prospective (non-randomized), and retrospective studies. Studies in which pharyngeal OSA surgery with or without nasal surgery was performed were included. However, studies in which nasal surgery alone was performed were excluded. In addition, studies were excluded if PAP therapy data, such as optimal pressure and/or compliance, were not clearly provided before and after surgery or if they lacked the data necessary for meta-analysis. Pharyngeal OSA surgery included tonsillectomy, UPPP and its variants, Pillar implants, tongue base surgery and its variants, genioglossus advancement, and radiofrequency ablation.

After two reviewers independently screened all titles and abstracts for candidate papers, we excluded clinical studies that were ineligible or irrelevant. There were no language restrictions in any articles reviewed in this study. We thoroughly reviewed the finally selected studies.

### 2.3. Data Extraction

We extracted data from the finally selected articles based on standardized forms. The data collected included the study design, the total number of subjects, age (years), sex (male:female), body mass index (kg/m^2^), the apnea-hypopnea index (AHI; events/h), surgical procedures, PAP therapy-related outcome measures, optimal pressure level (cm H_2_O), and usage time (h/night).

### 2.4. Quality Assessment

The risk of bias was assessed by using the STROBE tool (https://www.strobe-statement.org/, accessed on 26 September 2022). Five domains of bias, including selection, measuring exposure and outcome, controlling confound, sources of bias, and statistical method, were categorized as low, high, or unclear risk. The total quality of each study was defined as good, fair, or low. Two reviewers assessed the risk of bias in each included study independently, and disagreements were resolved by discussion with the other authors.

### 2.5. Statistical Analysis

The optimal levels and PAP usage time before and after pharyngeal OSA surgery were compared. For this, we collected the mean and standard deviation (SD) values of optimal levels and the usage time of PAP before and after pharyngeal surgery from the relevant studies. Heterogeneity was calculated with Cochran’s *Q* and *I^2^* tests. The *I^2^* test describes the rate of variation across studies because of heterogeneity rather than chance and ranges from 0 (no heterogeneity) to 100 (maximum heterogeneity). All results are reported with 95% confidence intervals (CIs), and all p-values were two-tailed. When significant heterogeneity among the outcomes was found (*I^2^* > 50), the random-effects model according to DerSimonian and Laird was used. This model assumes that the true treatment effects in the individual studies may be different from one another and that they are normally distributed. If the heterogeneity was not large (*I^2^* < 50), we planned to analyze it with a fixed-effect model. However, the effect model was not used due to the large heterogeneity of all results. We used a funnel plot and Egger’s test simultaneously to detect publication bias. Analyses were performed using Comprehensive Meta-Analysis V2 software (Biostat, Englewood, NJ, USA).

## 3. Results

**Figure 1:**
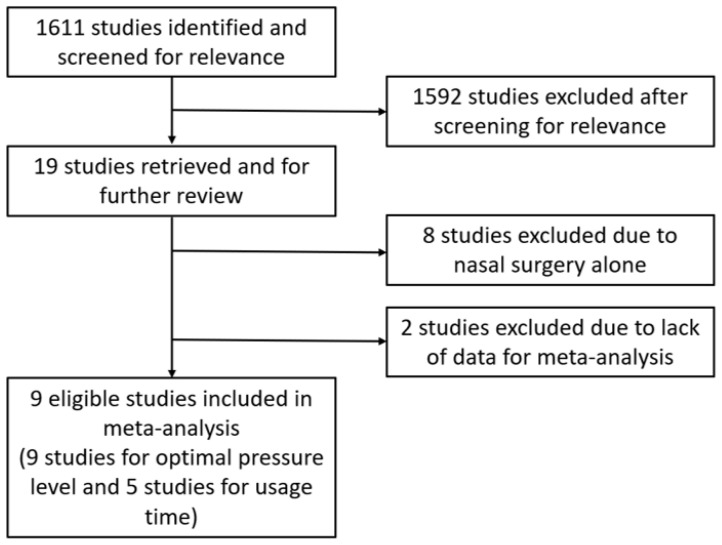
Flow diagram of the literature selection.

Figure 1 shows a flow diagram of the literature selection. After screening for relevance, 19 studies that investigated PAP therapy data, such as optimal pressure levels and/or usage time before and after surgery, were retrieved for further review [14,15,18,19,20,21,22,23,24,25,26,27,28,29,30,31,32,33,34,35,36,37]. We excluded eight studies evaluating the effect of nasal surgery alone on PAP therapy [14,19,20,21,22,23,24,25]. Two other studies were excluded due to the lack of data required for meta-analysis [15,18]. Finally, nine eligible studies were included in the meta-analysis (nine studies for optimal pressure levels and five studies for usage time) [26,27,28,29,30,31,32,33,34]. Table 1 presents the characteristics of the studies that met the inclusion criteria. Table 2 summarizes the comparison of PAP therapy, including optimal pressure and usage time before and after pharyngeal OSA surgery. All studies were judged to be fair for the risk of bias by combining judgments in the five domains.

### 3.1. Optimal PAP Level before and after Pharyngeal OSA Surgery

**Figure 2:**
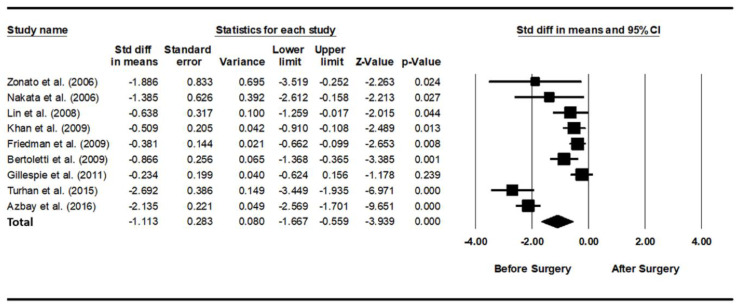
Forest plot for the effects of pharyngeal obstructive sleep apnea surgery on the optimal positive airway pressure level. The summed optimal PAP level was significantly lower after pharyngeal OSA surgery than before surgery. Std diff, standardized difference; CI, confidence interval [26,27,28,29,30,31,32,33,34].

**Figure 3:**
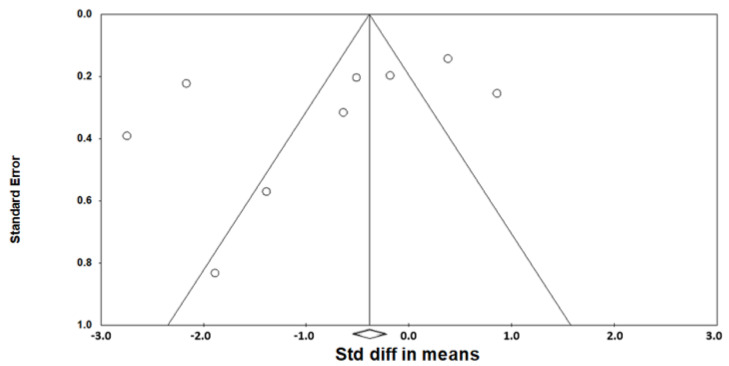
Funnel plot for the effects of pharyngeal obstructive sleep apnea surgery on the optimal positive airway pressure level. There was no publication bias (the Egger test *p*-value = 0.182). Std diff, standardized difference.

As heterogeneity was present among the studies (*Q*-value, 28.7; *p* < 0.001; *I^2^*, 86.1), a random-effects model was used. Figure 2 shows the forest plot for the effects of pharyngeal OSA surgery on optimal PAP levels. The summed optimal PAP level was significantly lower after pharyngeal OSA surgery than before surgery (standardized mean difference (SMD), −1.113; 95% CI, −1.667 to −0.559) [26,27,28,29,30,31,32,33,34]. Although the funnel plot looks slightly asymmetrical, we thought there was no publication bias because the Egger test *p*-value was 0.182 (Figure 3).

### 3.2. Usage Time of PAP before and after Pharyngeal OSA Surgery

**Figure 4:**
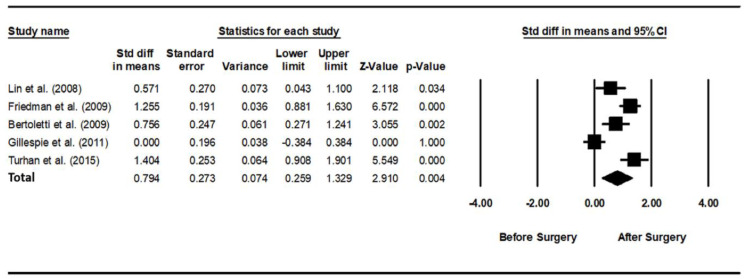
Forest plot for the effects of pharyngeal obstructive sleep apnea surgery on positive airway pressure usage time. The summed usage time of PAP increased significantly after pharyngeal OSA surgery compared to before surgery. Std diff, standardized difference; CI, confidence interval [28,30,31,32,33].

**Figure 5:**
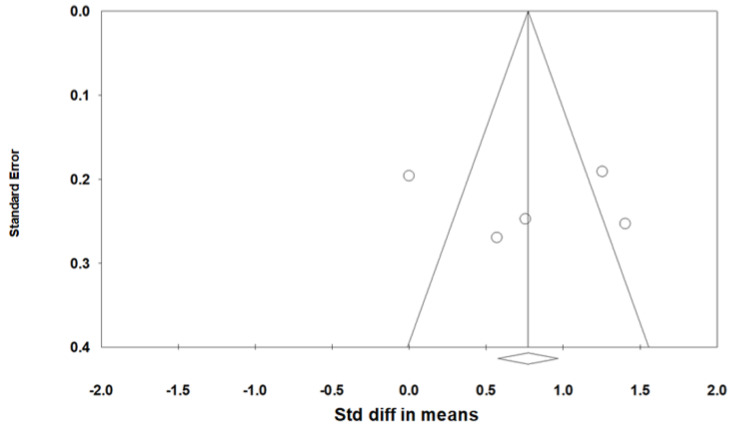
Funnel plot for the effects of pharyngeal obstructive sleep apnea surgery on positive airway pressure usage time. There was no publication bias (the Egger test *p*-value = 0.792). Std diff, standardized difference.

As heterogeneity was present among the studies (*Q*-value, 28.7; *p* < 0.001; *I^2^*, 86.1), a random-effects model was used. Figure 4 shows the forest plot for the effects of pharyngeal OSA surgery on usage time. The summed usage time of PAP increased significantly after pharyngeal OSA surgery compared to before surgery (SMD, 0.794; 95% CI, 0.259 to 1.329) [28,31,32,33]. The funnel plot looks symmetrical, and the Egger test *p*-value was 0.792, indicating no publication bias (Figure 5).

## 4. Discussion

There are several surgical indications for managing OSA [2,5]. Surgical modifications of the upper airway are usually performed for the purpose of directly improving respiratory disturbances during sleep. In addition, surgical therapy can be carried out as adjunctive management to alleviate the intolerance of other OSA treatments, such as PAP therapy. This study was designed to determine the effect of pharyngeal OSA surgery on PAP therapy, including optimal pressure levels, and compliance based on a meta-analysis. The results of the study demonstrated that optimal PAP levels decreased, and PAP usage time increased in patients with OSA after pharyngeal surgery.

Surgical outcomes may vary in patients with OSA depending upon the type of surgical procedure [35]. The results of numerous clinical studies have shown that isolated nasal surgery improved excessive daytime sleepiness and sleep-disordered breathing, such as snoring [36]. In contrast, whether nasal surgery alone statistically decreases the AHI is controversial [36,37]. A recent meta-analysis showed that isolated nasal surgery significantly improved the AHI in patients with OSA, but the alleviation of AHI was only slightly significant [37]. The outcomes of pharyngeal surgery for OSA are somewhat different from those of nasal surgery alone. For example, UPPP, one of the most representative pharyngeal surgeries, has been reported to have a surgical success rate of 35% to 70% when conducted randomly in patients with OSA [38,39]. According to our recent study assessing the AHI reduction ratio after oropharyngeal OSA surgery, such as UPPP, the postoperative AHI decreased by 30.4% to 74.1% based on an anatomy-based staging system [40]. The results of the study indicated that two different types of surgery, nasal surgery and pharyngeal surgery, had a similar effect on PAP treatment in patients with OSA despite different surgical sites and effects.

This meta-analysis comparing changes in optimal PAP levels before and after pharyngeal OSA surgery confirmed that the optimal pressure levels decreased from a mean ± standard error (SE) of 11.6 ± 0.4 to 9.3 ± 0.3 cm H_2_O. These outcomes are quite similar to earlier meta-analyses reports that isolated nasal surgery or upper airway surgery influenced optimal PAP levels in patients with OSA [16,17]. Camacho et al. [16] investigated the effect of nasal surgery alone on optimal PAP levels using meta-analysis. In their study, isolated nasal surgery included septoplasty, turbinoplasty, septoturbinoplasty, septorhinoplasty, and endoscopic sinus surgery [16]. The results of the meta-analysis of seven eligible studies established that the optimal pressure levels (mean ± SD) diminished from 11.6 ± 2.2 to 9.5 ± 2.0 cm H_2_O after nasal surgery alone [16]. Ayers et al. [17] evaluated the effect of upper airway surgery for OSA on optimal PAP levels based on a meta-analysis. In their meta-analysis of 11 eligible studies, upper airway surgery included isolated nasal surgery and various other types of pharyngeal surgery [17]. They found that the optimal pressure level reduced from a mean of 10.8 to 9.4 cm H_2_O in patients with OSA after upper airway surgery [17]. Although these two previous meta-analyses demonstrated that diverse surgical treatments play an important role in decreasing optimal PAP levels, the effect of pharyngeal OSA surgery on optimal pressure levels was not verified.

There are various PAP-related adverse effects, including mouth dryness, unintentional mask removal, skin irritation, air or mouth leak, pressure intolerance, mask claustrophobia, aerophagia (bloating), and nasal symptoms [11]. If these side effects are not addressed, PAP adherence could inevitably decrease. In particular, nasal obstruction or congestion leads to discomfort for patients with OSA because the nose is where the air generated from the PAP device comes into direct contact for the first time. A patient’s discomfort during PAP therapy can cause mouth leaks or unintentional mask removal, which diminishes PAP usage time [11]. There are two main methods to alleviate nasal obstructions, medical and surgical therapy. In general, surgical treatment is considered when medical management, including nasal irrigation, topical spray, and medication, is not effective. It is well-recognized that PAP adherence is improved in patients with OSA after nasal surgery alone [16]. Poirier et al. [25] examined the hypothesis that isolated nasal surgery enhanced PAP adherence in PAP-intolerant OSA patients with nasal obstructions and found that PAP usage time improved significantly from a mean ± SD of 0.5 ± 0.7 to 5.0 ± 2.4 h per night (*n* = 16) after nasal surgery (e.g., septoplasty and turbinoplasty) alone. In addition, the outcomes of the meta-analysis from four eligible papers demonstrated that PAP usage time increased by 0.62 h per night with a 95% CI of 0.22 to 1.01 after upper airway surgery, including isolated nasal surgery [17]. However, the effect of pharyngeal surgery on PAP adherence in patients with OSA has not yet been demonstrated based on a meta-analysis. Moreover, it has been argued that pharyngeal surgery such as UPPP may be associated with decreased PAP adherence [18]. This study found that PAP usage time increased from a mean ± SE of 4.2 ± 1.7 to 5.5 ± 0.5 h per night, comparing changes between preoperative and postoperative PAP usage. As a result, pharyngeal OSA surgery can indirectly enhance PAP adherence, as well as directly alleviate objective respiratory parameters, such as AHI, and subjective symptoms, such as excessive daytime sleepiness.

This meta-analysis had several limitations. There were few clinical studies with high-quality evidence in the analysis. Only one randomized controlled trial, three prospective studies, and five retrospective studies were included in the final analysis. Clinical investigations related to surgery have difficulties planning high-level evidence-based designs due to the nature of surgery. Nevertheless, further high-quality evidence studies are required. The study had heterogeneity in many aspects including the study design, sample size, differences in the surgical procedures, and characteristics of the populations. The results should be interpreted cautiously because the studies included in this meta-analysis were relatively small.

## 5. Conclusions

This meta-analysis demonstrated that pharyngeal surgery in patients with OSA decreased optimal PAP levels and increased PAP usage time. OSA should be regarded as a chronic disorder requiring long-term and comprehensive treatment. Although PAP is the main therapy for OSA, some patients with OSA are intolerant of PAP or fail PAP treatment. In these cases, pharyngeal OSA surgery can be considered an adjunct therapy to increase PAP adherence, even in patients who are not expected to be cured by surgery. The results of the study contribute to our understanding of the role of pharyngeal OSA surgery in PAP-intolerant patients.

## Figures and Tables

**Table 1:** Characteristics of the included studies.

References	Year	Level of Evidence (Study Design)	Total No. of Subjects	Age (Years)	Sex (M:F)	BMI (kg/m²)	AHI (Events/Hour)	Surgical Procedures	PAP Therapy Related Outcomes Measures
Zonato et al. [26]	2006	Level IV (retrospective)	17	49.0 ± 9.0	16:1	30.0 ± 4.0	38.0 ± 19.0	Tonsillectomy ± nasal surgery	Optimal PAP level
Nakata et al. [27]	2006	Level II-2 (prospective)	30	33.2 ± 6.8	28:2	30.7 ± 6.0	69.0 ± 28.4	Tonsillectomy	Optimal PAP level
Lin et al. [28]	2008	Level IV (retrospective)	16			34.1 ± 4.7	65.2 ± 49.2	Site-specific upper airway surgery (UPPP, pillar palatoplasty, GA, HMA, and repose tongue suspension)	Optimal PAP level PAP usage time
Khan et al. [29]	2009	Level IV (retrospective)	63	42.1 ± 13.9	51:12	34.9 ± 7.2	62.0 ± 35.4	UPPP ± tongue base surgery ± nasal surgery	Optimal PAP level
Friedman et al. [30]	2009	Level IV (retrospective)	52	43.1 ± 9.1	42:10	31.2 ± 5.0	63.2 ± 22.0	Multi-level surgery (UPPP, RFBOT, and nasal surgery)	Optimal PAP level PAP usage time
Bertoletti et al. [31]	2009	Level II-2 (prospective)	21	49.6 ± 11.2	16:5	31.4 ± 3.2	41.1 ± 5.8	Pillar palatal implants	Optimal PAP level PAP usage time
Gillespie et al. [32]	2011	Level I (RCT)	26	52.3 ± 10.3	22:4	34.7 ± 5.0	42.0 ± 21.0	Pillar palatal implants	Optimal PAP level PAP usage time
Turhan et al. [33]	2015	Level II-2 (prospective)	31	48 (31–66)	27:4	31.0 ± 2.4	44.7 ± 17.1	Modified tongue base suspension	Optimal PAP level PAP usage time
Azbay et al. [34]	2016	Level IV (retrospective)	67	47.0 ± 9.8	59:8	31.6 ± 4.2	45.0 ± 19.8	Modified UPPP + septoplasty ± modified tongue base suspension	Optimal PAP level

RCT, randomized controlled trial; M, male; F, female; BMI, body mass index; AHI, apnea-hypopnea index; UPPP, uvulopalatopharyngoplasty; GA, genioglossus advancement; HMA, hyoid myotomy and advancement; RFBOT, radiofrequency base of tongue reduction; PAP, positive airway pressure.

**Table 2:** Effects of pharyngeal obstructive sleep apnea surgery on positive airway pressure therapy, including optimal pressure and usage time.

References	Year	Final No. of Patients	Preoperative Optimal Pressure (cmH_2_O)	Postoperative Optimal Pressure (cmH_2_O)	*p* Value	Final No. of Patients	Preoperative Usage Time (Hours/Night)	Postoperative Usage Time (Hours/Night)	*p* Value
Zonato et al. [26]	2006	4	13.8 ± 1.5	10.4 ± 2.0	<0.05				
Nakata et al. [27]	2006	5	13.6 ± 2.5	10.6 ± 1.3	<0.05				
Lin et al. [28]	2008	12	11.5 ± 3.7	9.4 ± 2.6	<0.05	16	4.1 ± 2.4	5.5 ± 2.5	<0.05
Khan et al. [29]	2009	27	9.7 ± 3.0	8.3 ± 2.4	<0.05				
Friedman et al. [30]	2009	52	10.6 ± 2.1	9.8 ± 2.1	<0.05	49	0.02 ± 0.14	3.2 ± 2.6	<0.001
Bertoletti et al. [31]	2009	21	11.2 ± 1.6	9.3 ± 2.5	<0.05	21	5.7 ± 0.9	6.3 ± 0.6	<0.05
Gillespie et al. [32]	2011	26	10.9 ± 2.7	10.3 ± 2.4	NS	26	6.0 ± 2.5	6.0 ± 2.4	NS
Turhan et al. [33]	2015	31	12.6 ± 1.6	8.0 ± 1.8	<0.001	31	5.3 ± 0.8	6.5 ± 0.9	<0.001
Azbay et al. [34]	2016	67	11.8 ± 1.4	9.0 ± 1.2	<0.001				

NS, not significant.

## Data Availability

The datasets used and/or analyzed during the current study available from the corresponding author on request.
